# Public knowledge, attitudes, and practices toward heat stroke in Ningbo, China: a cross-sectional study

**DOI:** 10.3389/fpubh.2025.1659132

**Published:** 2025-09-25

**Authors:** Wei Zhang, Yangrong Feng, Shuren Chai, Zheng Yang, Jing Dong

**Affiliations:** ^1^Department of Emergency, Ningbo Municipal Hospital of Traditional Chinese Medicine (TCM), Affiliated Hospital of Zhejiang Chinese Medical University, Ningbo, Zhejiang, China; ^2^Department of Respiratory, Ningbo Municipal Hospital of Traditional Chinese Medicine (TCM), Affiliated Hospital of Zhejiang Chinese Medical University, Ningbo, Zhejiang, China; ^3^Department of Emergency, The First Affiliated Hospital of Ningbo University, Ningbo, Zhejiang, China

**Keywords:** heat stroke, knowledge, attitude, practice, cross-sectional study

## Abstract

**Background:**

Heat stroke, a severe heat illness with high mortality rates, is increasingly prevalent due to rising temperatures. This study aimed to assess public knowledge, attitudes, and practices (KAP) regarding heat stroke.

**Methods:**

We conducted a cross-sectional study in Ningbo City from December 10, 2023, to March 21, 2024. Data were collected using a structured, self-administered electronic KAP questionnaire designed to assess knowledge, attitudes, and practices regarding heat stroke. Statistical analysis was performed using SPSS 27.0 and AMOS 26.0, including descriptive statistics, correlation analysis, logistic regression, and structural equation modeling (SEM).

**Results:**

The study enrolled 467 participants, of whom 396 (84.81%) were aged between 18 and 45 years, and 294 (62.96%) were female. The median scores for knowledge, attitude, and practice exceeded 80% of their respective total score dimensions, at 26(range: 23–26), 35(range: 33–35), and 40 points (range: 35–44). Structural equation modeling (SEM) showed direct impacts of knowledge on attitude (*β* = 0.276, *p* = 0.009) and direct influences of both knowledge (*β* = 0.133, *p* = 0.012) and attitude (*β* = 0.431, *p* = 0.012) on practice. Moreover, knowledge indirectly affected practice through its impact on attitude (*β* = 0.119, *p* = 0.008). Multivariate logistic regression found that attitude score (OR = 1.555, *p* < 0.001), BMI under 18(OR = 4.135, *p* = 0.024), marital status (married: OR = 0.433, *p* = 0.001), and Bachelor’s degree and below (High school/vocational school and below: OR = 0.294, *p* = 0.013; junior college: OR = 0.340, *p* = 0.018; bachelor’s degree: OR = 0.410, *p* = 0.027) correlated with practice scores.

**Conclusion:**

While the Ningbo public showed sufficient general understanding of heat stroke, the study found serious gaps in understanding specific risk factors and preventive measures. The findings suggest tailored health education programs, particularly for young people and less educated populations, to promote community-wide awareness and prevention.

## Introduction

Heat stroke, the most severe form of heat illness, emerges as a critical public health concern globally due to the increasing incidence of heat waves. This condition, characterized by a core body temperature exceeding 40 °C, stems from an imbalance between the body’s heat production and dissipation. Such imbalances occur due to exposure to high ambient temperatures and/or intense physical activity. The resulting heat load overwhelms the body’s cooling mechanisms, potentially initiating heat stroke in environments where heat cannot be effectively dissipated (1).

Clinically, heat stroke manifests through severe central nervous system abnormalities, including altered mental status, convulsions, or coma, and may precipitate life-threatening multi-organ damage. The spectrum of heat stroke includes classic heat stroke, typically triggered by environmental heat during summer waves, and exertional heat stroke, often linked to physical exertion (2–4).

Epidemiologically, the incidence of classic heat stroke ranges from 17.6 to 26.5 cases per 100,000 during heat waves, with a mortality rate among hospitalized patients between 14 and 65%. Conversely, exertional heat stroke comprises 8.6 to 18.0% of heat-induced ailments (5).

As the most extreme heat-related illness, heat stroke can lead to dysfunction in the central nervous system, widespread organ failure, cardiovascular damage, and even death. This underscores the urgent need for enhanced public health strategies and interventions to mitigate the impact of heat waves and prevent heat stroke.

The Knowledge, Attitude, and Practice (KAP) model asserts that individual behaviors are influenced by one’s knowledge and attitudes. This framework is critical in public health for elucidating health-related behaviors, particularly through the use of KAP surveys that assess both knowledge and risk perception (6–8). Understanding public perceptions and responses to heat stroke is essential for developing effective prevention and education strategies. These strategies are designed to bridge knowledge gaps and enhance preparedness, thereby reducing the potentially severe impacts on health systems during peak heat periods. In China, public health research on urban heat waves is gaining increasing attention. For example, one study conducted in the extremely hot-humid city of Chongqing explored residents’ perceptions, physiological and psychological impacts, adaptive awareness, and broader issues such as climate justice (9). While such research provides a valuable macro-level perspective, there remains a lack of refined KAP studies that focus on the interaction pathways between knowledge, attitudes, and practices in climate-specific cities—such as Ningbo, a subtropical coastal city with dense population and prolonged humid summers.

In China, where the climate is marked by minimal regional temperature variations and prolonged periods of consistent high temperatures, this research becomes even more crucial. Some regions in China experience summer temperatures that exceed 39 °C (5). Such extreme conditions underscore the importance of tailored public health interventions to safeguard particularly vulnerable populations, who are often disproportionately affected by heat waves and may lack the resources or knowledge necessary for effective self-protection. While international studies have explored heat stroke KAP, research within China remains limited, particularly focusing on the unique context of subtropical coastal cities which are characterized by high-humidity, high-temperature summers and a dense urban population. These cities, like Ningbo, represent a critical area for public health intervention.

This study, therefore, focuses on Ningbo, a representative subtropical coastal city in China, to assess the public’s KAP regarding heat stroke. The findings aim to provide evidence for developing targeted health education strategies for Ningbo and other cities with similar climatic and socioeconomic characteristics, thereby contributing to a nuanced understanding of heat stroke preparedness in urban China.

## Materials and methods

### Study design and participants

This cross-sectional study was conducted in Ningbo City from December 10, 2023, to March 21, 2024. Participants were voluntary members of the public, aged 18 years and older, possessing normal cognitive function and without communication barriers. This study was approved by the Ethic Committee of Ningbo Municipal Hospital of Traditional Chinese Medicine (TCM), Affiliated Hospital of Zhejiang Chinese Medical University (KYSL-2023-010). Written informed consent was secured from all participants prior to the distribution of questionnaires, which was facilitated electronically; only those who consented were permitted to complete the questionnaire.

### Questionnaire quality control

The electronic questionnaire was developed using Questionnaire Star, which facilitated the creation of an online survey and the generation of a QR code. Participants accessed the questionnaire by scanning this QR code. To maintain the integrity and completeness of the responses, each IP address was restricted to a single submission, and completion of all items was mandatory. The research team rigorously reviewed the completeness, internal consistency, and logical coherence of all submitted questionnaires.

### Questionnaire introduction

The questionnaire was developed by the research team after a comprehensive literature review of existing KAP studies on heat-related illnesses and in accordance with guidelines from the Chinese Center for Disease Control and Prevention. The initial draft was reviewed for content validity by two critical care experts. Following their feedback, a pilot test was conducted with 33 participants. The final version of the questionnaire was structured into four sections: demographic data (including education level, gender, occupation, etc.), and dimensions assessing knowledge, attitude, and practice (Appendix).

The knowledge dimension comprised 14 questions covering definitions, risk factors, symptoms, prevention, and treatment of heat stroke. The final scoring system for the knowledge section (2 points for correct, 1 for unclear/incorrect) was designed to differentiate definitive knowledge from uncertainty, a method used in similar public health surveys (10), allowing for a total score ranging from 14 to 28. The attitude dimension contained 7 questions evaluating perceptions of heat stroke’s harm, the necessity of prevention, and self-efficacy in managing heat stroke, utilizing a five-point Likert scale from very positive (5 points) to very negative (1 point). This section had a potential score range of 7 to 35. The practice dimension included 9 questions on preventive measures against heat stroke, also scored on a five-point Likert scale from always (5 points) to never (1 point), with scores ranging from 9 to 45. Scores above 80% of the maximum in each section were considered indicative of adequate knowledge, a positive attitude, and proactive practice (11).

### Sample size calculation

The calculation of the sample size was performed using the following formula:

n=(Z_(1−α/2)/δ)^2×p×(1−p) .

Here, n represents the sample size, and p was assumed to be 0.5 to maximize the sample size. The significance level α, or Type I error, was set at 0.05, resulting in Z (1-α/2) = 1.96. The standard error (*δ*) was assumed to be 0.05. Accounting for an anticipated questionnaire response rate of 90%, the goal was to collect at least 430 completed questionnaires. This approach is widely used in cross-sectional KAP studies to ensure sufficient power and representation (12).

### Statistical analysis

Data analysis was conducted using SPSS 27.0 (IBM, Armonk, New York, United States) and Amos 26.0 (IBM, Armonk, New York, United States). Descriptive analysis was performed on the demographic data of the respondents and the scores across each dimension. For continuous data, normality tests were first conducted. If the data were normally distributed, they were presented as means and standard deviations (SD); if not, medians along with the 25th and 75th percentiles were used. Categorical data, including demographic characteristics and responses to each question, were expressed as n (%). For comparisons among continuous variables, the t-test was employed for normally distributed data between two groups, and the Wilcoxon Mann–Whitney test was used for non-normally distributed data. For comparisons involving three or more groups, ANOVA was utilized for normally distributed variables with uniform variance, while the Kruskal-Wallis test was applied to non-normally distributed data. Correlation analysis was performed based on the distribution of the data: Pearson correlation coefficients were calculated for normally distributed data, and Spearman correlation coefficients were used for non-normally distributed data. Practice scores served as the dependent variable in both univariate and multivariate regression analyses to explore the associations between demographic data and scores across dimensions. A threshold of 80% of the total practice score, with scores above 38 indicating positive practice, was set. Variables with *p* < 0.01 in the univariate analysis were included in the multivariate regression. All *p*-values were reported to three decimal places, and a two-sided *p*-value less than 0.05 was considered statistically significant. To ensure the psychometric properties of the KAP scales, Confirmatory Factor Analysis (CFA) was first conducted for each dimension (knowledge, attitude, and practice) to evaluate their measurement model validity. Subsequently, Structural Equation Modeling (SEM) was utilized to analyze the relationships between the dimensions of the questionnaire and to explore the mediating role of attitude in the relationship between knowledge and practice.

## Results

### Demographic characteristics

A total of 502 participants were initially included in the study. After the exclusion of anomalous data, 467 cases remained valid for analysis. The overall Cronbach’s alpha for the feedback scale in the formal experiment was 0.896, with the specific dimensions scoring as follows: knowledge at 0.842, attitude at 0.908, and practice at 0.886. The KMO measure verified sampling adequacy with a value of 0.870. In addition, we conducted confirmatory factor analyses (CFA) for the knowledge, attitude, and practice scales (see Supplementary Figure S1 for the CFA model diagram). The CFA results indicated acceptable construct validity across all three dimensions, with good fit indices (χ^2^/df = 4.394, CFI = 0.837, TLI = 0.818, IFI = 0.838, RMSEA = 0.085). The factor loadings for each item were all statistically significant (*p* < 0.05), further supporting the reliability and validity of the measurement model (Supplementary Table S1).

Out of 467 participants, 396 (84.81%) were aged 18–45 years, 294 (62.96%) were females, with height of 163 (160–170) cm, weight of 58 (52–70) kg, and 292 (62.53%) had a BMI in the range of 18–24 kg/m^2^. Meanwhile, 256 (54.82%) had Bachelor’s degree, 35 (7.49%) had occupations involving working in high-temperature environments, and 21 (4.50%) had a history of heat stroke/severe heat exhaustion. The median scores of participants’ knowledge, attitudes, and practices (p25 to p75) were 26 (23 to 26), 35 (33 to 35), and 40 (35 to 44) above 80% of the highest scores (K: 28, A: 35, P: 45), respectively. The knowledge scores varied from participants with different age (*p* = 0.003), education (*p* < 0.001), and history of heat stroke/severe heat exhaustion (*p* = 0.036). The attitude scores varied from participants with different age (*p* = 0.016), gender (*p* = 0.019), and presence of underlying diseases (*p* = 0.004). The practice scores varied from participants with different age (*p* = 0.003), gender (*p* < 0.001), marital Status (*p* = 0.002), residence (*p* = 0.020), education (*p* = 0.011), occupation (*p* = 0.010), history of heat stroke/severe heat exhaustion (*p* = 0.037), and presence of underlying diseases (*p* = 0.012; Tables 1, 2).

**Table 1:** Demographic characteristics of participants (e.g., age, gender, BMI, height, weight, education level, occupation, etc.).

Demographic characteristic	N (%)
*N* = 467	
Total Score	
Age	
18–45 years	401 (85.87%)
46–69 years	66 (14.13%)
Height	163 (160–170)
Weight	58 (52–70)
BMI	
Less than 18	30 (6.42%)
18–24	292 (62.53%)
Greater than or equal to 24	145 (31.05%)
Gender	
Male	173 (37.04%)
Female	294 (62.96%)
Marital Status	
Married	134 (28.69%)
Other	333 (71.31%)
Residence	
Urban	414 (88.65%)
Rural	53 (11.35%)
Education Level	
High school/technical school and below	68 (14.56%)
Junior college	86 (18.42%)
Bachelor’s degree	256 (54.82%)
Master’s degree or above	57 (12.21%)
Occupation	
Occupations involving working in high-temperature environments (e.g., sanitation workers, traffic police, firefighters, athletes/sports coaches, construction workers)	35 (7.49%)
Other occupations not involving working in high-temperature environments	432 (92.51%)
Average disposable income per capita in the family per year	
Less than 50,000	143 (30.62%)
50,000 or more	324 (69.38%)
History of heat stroke/severe heat exhaustion	
Yes	21 (4.50%)
No	446 (95.50%)
Presence of diabetes, cardiovascular disease, or other underlying diseases	
With underlying diseases	50 (10.71%)
Without any diseases	417 (89.29%)

**Table 2:** KAP score differences across subgroups based on demographic characteristics.

Demographic characteristic	Knowledge, median (P25-P75)	*p*	Attitude, median (P25-P75)	*p*	Practice, median (P25-P75)	*p*
*N* = 467						
Total Score	26 (23–26)		35 (33–35)		40 (35–44)	
Age		0.003		0.016		0.003
18–45 years	24 (23–27)		34 (33–35)		38.5 (36–44)	
46–69 years	26 (22–27)		35 (29–35)		41 (32–41.25)	
Height						
Weight						
BMI		0.553		0.118		0.084
Less than 18	24.5 (22.75–26)		35 (34–35)		41.5 (38–44)	
18–24	25.5 (23.25–26)		35 (33–35)		40 (35–44)	
Greater than or equal to 24	26 (22–27)		35 (31–35)		39 (35–43)	
Gender		0.158		0.019		<0.001
Male	26 (22–27)		35 (31–35)		39 (32–42)	
Female	26 (24–26)		35 (33–35)		41 (36–44)	
Marital Status		0.188		0.659		0.002
Married	25 (23–26)		35 (32–35)		38 (32–43)	
Other	26 (24–26)		35 (33–35)		40 (36–44)	
Residence		0.376		0.819		0.020
Urban	25 (23–26)		35 (33–35)		40 (35–45)	
Rural	26 (23.5–26.5)		35 (30–35)		36 (35–44.6)	
Education Level		<0.001		0.156		0.011
High school/technical school and below	23 (20–26)		35 (29.25–35)		38.5 (32–42.75)	
Junior college	25 (24–27)		35 (33–35)		38 (36–44.25)	
Bachelor’s degree	26 (23–26)		35 (33–35)		40 (35–44)	
Master’s degree or above	26 (25–27)		35 (33–35)		42 (38–44)	
Occupation		0.074		0.852		0.010
Occupations involving working in high-temperature environments	26 (25–27)		35 (33–35)		38 (29–42)	
Other occupations not involving working in high-temperature environments	25 (23–26)		35 (33–35)		40 (35–44)	
Average disposable income per capita in the family per year		0.203		0.252		0.568
Less than 50,000	26 (23–27)		35 (33–35)		39 (35–44)	
50,000 or more	25 (23–26)		35 (33–35)		40 (36–44)	
History of heat stroke/severe heat exhaustion		0.036		0.607		0.037
Yes	26 (25–27)		35 (30–35)		40 (31.5–39.5)	
No	25 (23–26)		35 (33–35)		37 (35–44)	
Presence of diabetes, cardiovascular disease, or other underlying diseases		0.110		0.044		0.012
With underlying diseases	24 (22–26)		33 (30–35)		38.5 (32–40)	
Without any diseases	26 (23–26.5)		35 (33–35)		41 (36–44)	

### Knowledge assessment

The distribution of knowledge dimensions among participants indicates several critical misunderstandings that have significant clinical implications. Notably, a substantial portion of respondents believe that heat stroke cannot occur during exercise in cool environments (K6) with 43.25% incorrectly denying its possibility, and 17.56% uncertain, showcasing a critical gap in understanding the conditions under which heat stroke can occur. Additionally, there is considerable uncertainty regarding the use of medications like antipyretics in treating heat stroke, with 26.98% of participants uncertain about its necessity (K12; Table 3).

**Table 3:** Knowledge dimension of the participants.

Item	N (%)		
	Yes	No	Uncertain
1. heat stroke, also known as severe heat stroke, is divided into classic heat stroke and exertional heat stroke, often occurring in humid and hot conditions.	302 (64.67%)	13 (2.78%)	152 (32.55%)
2. Classic heat stroke is due to impaired heat dissipation caused by dysfunction in temperature regulation in high-temperature environments.	349 (74.73%)	8 (1.71%)	110 (23.55%)
3. Classic heat stroke mainly occurs in the older adults, children, and individuals with underlying diseases.	364 (77.94%)	41 (8.78%)	62 (13.28%)
4. Inadequate intake of water to replenish fluid loss caused by sweating can lead to dehydration, which is an important factor in the occurrence of non-exertional heat stroke.	366 (78.37%)	16 (3.43%)	85 (18.20%)
5. Exertional heat stroke typically occurs in previously healthy young individuals who engage in vigorous exercise in high ambient temperatures and humidity.	367 (78.59%)	22 (4.71%)	78 (16.70%)
6. heat stroke cannot occur during exercise in cool environments.	183 (39.19%)	202 (43.25%)	82 (17.56%)
7. Symptoms of heat stroke can include dizziness, nausea, muscle cramps, and confusion.	426 (91.22%)	5 (1.07%)	36 (7.71%)
8. In clinical settings, heat stroke can manifest as central nervous system dysfunction and multi-organ system failure.	417 (89.29%)	5 (1.07%)	45 (9.64%)
9. If not promptly treated, heat stroke can result in death.	428 (91.65%)	3 (0.64%)	36 (7.71%)
10. The core body temperature of heat stroke patients can rise to over 40 °C.	419 (89.72%)	4 (0.86%)	44 (9.42%)
11. Rapid, effective, and sustained cooling is the primary treatment for heat stroke.	422 (90.36%)	6 (1.28%)	39 (8.35%)
12. The use of medications such as antipyretics to reduce fever may be necessary in the treatment of heat stroke.	286 (61.24%)	55 (11.78%)	126 (26.98%)
13. heat stroke patients may experience involuntary muscle spasms.	366 (78.37%)	7 (1.50%)	94 (20.13%)
14. Common herbs such as chrysanthemum, honeysuckle, lotus leaf, peppermint, patchouli, and pogostemon cablin have heat-clearing and heat stroke-preventing effects to some extent.	369 (78.37%)	3 (0.64%)	95 (20.34%)

### Attitudes towards heat stroke

When it comes to related attitudes, 81.37% strongly agreed that public places should be provided with adequate heat stroke prevention facilities to reduce the risk of heat stroke (A2), and 79.66% strongly agreed that it is necessary to focus on educating high-risk groups for heat stroke such as the older adults, children, and workers about heat stroke (A5). In addition, 72.81% were very confident in preventing heat stroke (A7; Supplementary Figure S2A).

### Preventive practices

The distribution of practice dimensions regarding heat stroke prevention among participants shows varying levels of engagement in proactive measures. A significant gap in consistent preventive practices is evident with only 37.69% of participants always ensuring that their homes are stocked with cooling items such as chrysanthemum and honeysuckle during hot summers, and a further 24.20% doing so often (P9). This indicates a considerable portion of the population might not be adequately prepared to manage heat effectively. Additionally, only 59.96% of respondents always educate their family and friends about heat stroke prevention (P8), suggesting a need for broader community education efforts. The data also reveals a strong adherence to immediate response measures, with 66.17% always seeking medical help when experiencing symptoms of heat stroke (P7; Supplementary Figure S2B).

### Correlation and regression analysis

Correlation analysis indicated significant positive correlations between knowledge and attitude (*r* = 0.225, *p* < 0.001), as well as practice (*r* = 0.225, *p* < 0.001). Meanwhile, there was also correlation between attitude and practice (*r* = 0.508, *p* < 0.001; Table 4).

**Table 4:** Correlation analysis of KAP scores.

Variable	Knowledge	Attitude	Practice
Knowledge	1		
Attitude	0.225 (*p* < 0.001)	1	
Practice	0.225 (*p* < 0.001)	0.508 (*p* < 0.001)	1

Multivariate logistic regression showed that attitude score (OR = 1.555, 95% CI: [1.404–1.724], *p* < 0.001), BMI less than 18 (OR = 4.135, 95% CI: [1.202–14.223], *p* = 0.024), married (OR = 0.433, 95% CI: [0.258–0.726], *p* = 0.001), High school/vocational school and below (OR = 0.294, 95% CI: [0.112–0.775], *p* = 0.013), junior college education (OR = 0.340, 95% CI: [0.139–0.832], *p* = 0.018), Bachelor’s degree (OR = 0.410, 95% CI: [0.186–0.906], *p* = 0.027) were independently associated with practice (Supplementary Table S2).

**Figure 1:**
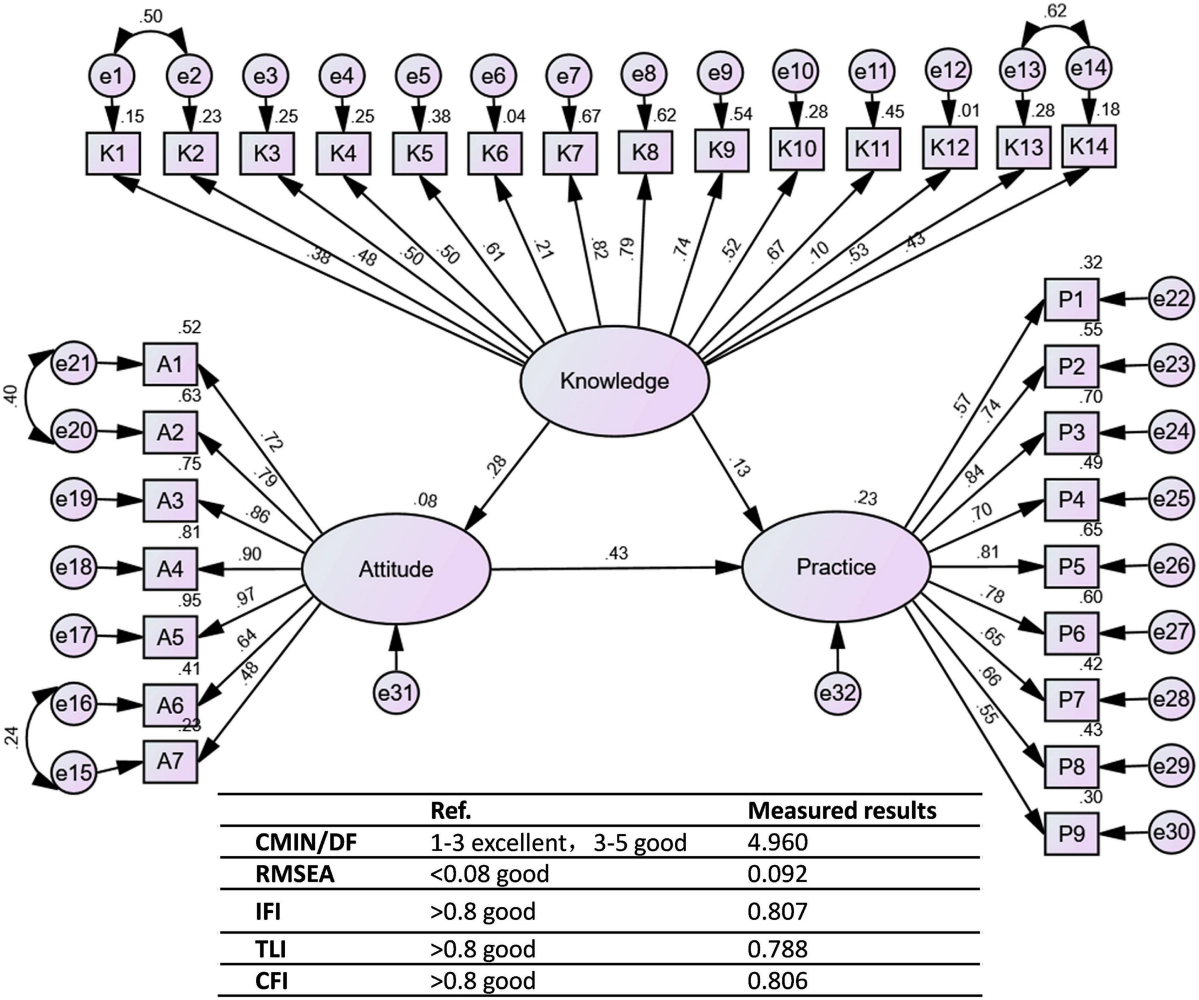
The structural equation model (SEM). Rectangles represent observed variables (KAP dimension scores), ellipses represent latent attitude variables, and circles represent residual terms. The number on the arrow is the normalized path factor (*β*), whose significance (*p*-value) indicates the strength and direction of the relationship.

To further explore the interplay between knowledge, attitude, and practice, a structural equation model was constructed (Figure 1). Regarding the overall Structural Equation Model (SEM) fit, some indices such as CMIN/DF (4.960) and RMSEA (0.092) were suboptimal, while other indices (CFI = 0.806, TLI = 0.788, IFI = 0.807) indicated a partially acceptable model fit, suggesting a usable structure despite some limitations. Supplementary Table S3 also shows the path coefficients and factor loads in the structural equation model. Bootstrap analysis of mediating effect significance test showed that knowledge directly affected attitude (*β* = 0.276, *p* = 0.009), knowledge (*β* = 0.133, *p* = 0.012) and attitude (*β* = 0.431, *p* = 0.012) directly affected practice, and knowledge indirectly affected practice through attitude (*β* = 0.119, *p* = 0.008; Table 5; Figure 1), suggesting that knowledge enhances preventive practices partly by shaping a more positive attitude.

**Table 5:** Bootstrap analysis of mediating effect significance test for the final mode.

Model paths	Standardized direct effects (95%CI)	*p*	Standardized indirect effects (95%CI)	*p*
Knowledge→Attitude	0.276 (0.140–0.409)	0.009		
Knowledge→Practice	0.133(0.024–0.262)	0.012	0.119 (0.066–0.178)	0.008
Attitude→Practice	0.431 (0.303–0.548)	0.012		

## Discussion

The study revealed that the public in Ningbo City possesses adequate knowledge, maintains positive attitudes, and engages in effective practices concerning heat stroke prevention and management. Given the significant influence of knowledge and attitudes on practices, it is recommended that public health interventions focus on enhancing educational programs to further improve the community’s response to heat stroke, particularly targeting high-risk demographics identified by the study.

Our analysis confirmed the interrelationship among KAP regarding heat stroke prevention, as evidenced by significant positive correlations and SEM. Knowledge was shown to positively influence attitudes and practices, and attitudes also significantly affected practices. This interconnection suggests that enhancing public knowledge about heat stroke can be an effective strategy in improving overall community preparedness and response (13, 14).

The study also highlighted significant variations in KAP regarding heat stroke prevention among different demographic groups, with particular disparities. Notably, younger participants (aged 18–45) demonstrated slightly lower knowledge and practice scores compared to older individuals, showing that younger age was associated with lower practice engagement. This may reflect a generational difference in exposure to and prioritization of heat stroke prevention information, suggesting a need for targeted educational campaigns aimed at younger populations (15, 16).

Education level emerged as a significant predictor across all KAP dimensions, with higher education correlating with better outcomes. This finding aligns with previous research indicating that higher educational attainment enhances health literacy, which in turn promotes better health behaviors (17, 18). The multivariate analysis reinforces this, with significant odds ratios indicating that higher educational levels, including junior college, bachelor’s degree, and master’s degree or above, are strongly associated with improved practices. An unexpected finding of this study was that married individuals had lower practice scores compared with unmarried participants (OR = 0.433). Although the present study was not designed to explore the underlying causes, possible explanations include greater family and work responsibilities limiting personal preventive actions, or a “diffusion of responsibility” within households where individuals assume that other members will take preventive measures. This observation warrants further investigation in future studies, for example through qualitative interviews or targeted quantitative surveys, to clarify the social and family dynamics behind this pattern (19).

Moreover, significant differences were noted in practices based on gender, with females engaging more frequently in heat stroke prevention practices than males. This could be linked to a generally higher level of health-conscious behavior observed among women in various settings (20, 21). Interestingly, participants with a history of heat stroke or severe heat exhaustion displayed better knowledge and practices. This could be due to their direct experience with the condition, enhancing their perception of its seriousness and triggering more vigilant self-protective behaviors (6). Individuals with chronic diseases showed lower practice scores and also lower knowledge scores, potentially reflecting an overarching burden of disease management that may limit capacity or motivation to adopt additional preventive measures (22, 23).

Moreover, we observed substantial gaps in the public’s understanding and practical response to heat stroke, despite generally positive attitudes toward the seriousness of the condition and the need for preventive measures. Notably, there was significant uncertainty among participants about heat stroke’s occurrence during exercise in cooler environments and the effectiveness of traditional remedies. To address these deficiencies, health authorities could leverage digital and community outreach platforms to clarify misconceptions about the conditions under which heat stroke can occur (24). Furthermore, embedding heat stroke prevention education into school curricula and workplace training could ensure that younger and working-age populations are better equipped to handle heat-related risks (25, 26). Our findings suggest that public health policies in Ningbo should move beyond general awareness campaigns. For instance, given the misconception that heat stroke cannot occur in cool environments, educational materials should explicitly feature real-life scenarios such as strenuous indoor exercise or hiking on a cool but humid day. Additionally, since lower educational attainment was significantly associated with poorer heat stroke prevention practices, there is a need for community-based, visual-first health education programs. These should be disseminated through familiar platforms like community centers, local television, and social media apps such as WeChat, using infographics and video content rather than text-heavy brochures to ensure accessibility and engagement among less-educated populations (27, 28). While this study underscores the importance of strengthening health education to modify individual behavior, relying solely on personal efforts is insufficient. Structural and environmental interventions are equally critical. Individual preventive actions can only be effectively sustained within supportive environments. For example, previous research has shown that through scientifically designed community planning—such as developing cool, walkable, and cyclable transportation systems—residents’ exposure to urban heat can be substantially reduced. This represents a promising approach for building heat-resilient cities (29). Therefore, we recommend that public health departments collaborate with urban planning, transportation, and landscaping sectors to jointly promote the construction of climate-adaptive communities. In addition to educational efforts, enhancing public health infrastructure by creating heat-relief zones with adequate hydration stations and shaded areas could materially support the community, particularly in public and communal spaces (30). Tailoring interventions to high-risk groups—such as the older adults, children, and outdoor workers—by providing specific guidance and heat safety kits could further strengthen community resilience. These kits could include items such as water bottles, electrolyte packets, and educational materials on recognizing and responding to heat stroke symptoms (31, 32). Encouraging community-led health promotion, where community members educate and support each other in adopting preventive practices, can reinforce positive behaviors and foster a supportive network (33, 34).

This study has several limitations that should be considered when interpreting the results. First, our sample did not specifically target high-risk populations such as the older adults (>69 years) and children, who are physiologically more vulnerable to heat stroke. The sample also contained a high proportion of younger adults and females, which may have introduced bias and limited the generalizability of the results. Specifically, younger adults (85.87% aged 18–45) and females (62.96%) were overrepresented, which may further restrict the applicability of the findings to high-risk groups such as the older adults. Future research should employ stratified sampling methods to ensure a more balanced representation of older adults participants, males, and other vulnerable groups, and should also develop tailored questionnaires or observational tools to capture their specific knowledge and behavioral needs regarding heat stroke prevention. Second, the cross-sectional design precludes the ability to infer causality between knowledge, attitudes, and practices related to heat stroke. Third, the data were collected during winter and spring rather than in the peak summer months, which may have reduced the reported levels of awareness and preventive practices, particularly those that are season-specific, such as stocking cooling items or adjusting daily routines during hot weather. Future studies should include follow-up surveys during peak summer periods to capture these seasonal behaviors more accurately. Fourth, the sample was drawn exclusively from Ningbo City, which may limit the generalizability of the findings to other geographical areas with different climate conditions and public health infrastructures. Finally, the self-reported nature of the data may introduce response bias, as participants might overestimate their knowledge or practices concerning heat stroke prevention. In addition, while confirmatory factor analysis (CFA) for the measurement model showed acceptable fit with some indices, its RMSEA value of 0.085 was slightly above the conventional good fit threshold of 0.08, suggesting minor room for improvement in the measurement model. Furthermore, the overall Structural Equation Model (SEM) fit indices did not fully reach the recommended thresholds (CMIN/DF = 4.960 and RMSEA = 0.092 were suboptimal). Although we retained all items to preserve content validity and theoretical completeness, these limitations in model fit suggest that the interpretation of specific path coefficients should be approached with appropriate caution. Future studies should test and refine the scale with larger and more diverse samples to achieve stronger psychometric performance.

## Conclusion

In conclusion, the study revealed that the public possesses adequate knowledge, maintains positive attitudes, and engages in appropriate practices concerning heat stroke, indicating an effective transmission of health information on this critical issue. These findings suggest the importance of continued educational interventions that enhance understanding and attitudes toward heat stroke, particularly focusing on targeted age groups and educational backgrounds to optimize preventive practices across the community.

## Data Availability

The original contributions presented in the study are included in the article/Supplementary material, further inquiries can be directed to the corresponding authors.

## Supplementary material

SUPPLEMENTARY FIGURE S1 Confirmatory factor analysis model.

SUPPLEMENTARY FIGURE S2 Participants' attitude, practice dimension of each item option distribution. **(A)** attitude; **(B)** practice.
