# Supplementary figures and images for: Public knowledge, attitudes, and practices toward heat stroke in Ningbo, China: a cross-sectional study
Source: Front Public Health. 2025 Sep 25;13:1659132. doi: 10.3389/fpubh.2025.1659132 (PMC12507894; doi:10.3389/fpubh.2025.1659132)

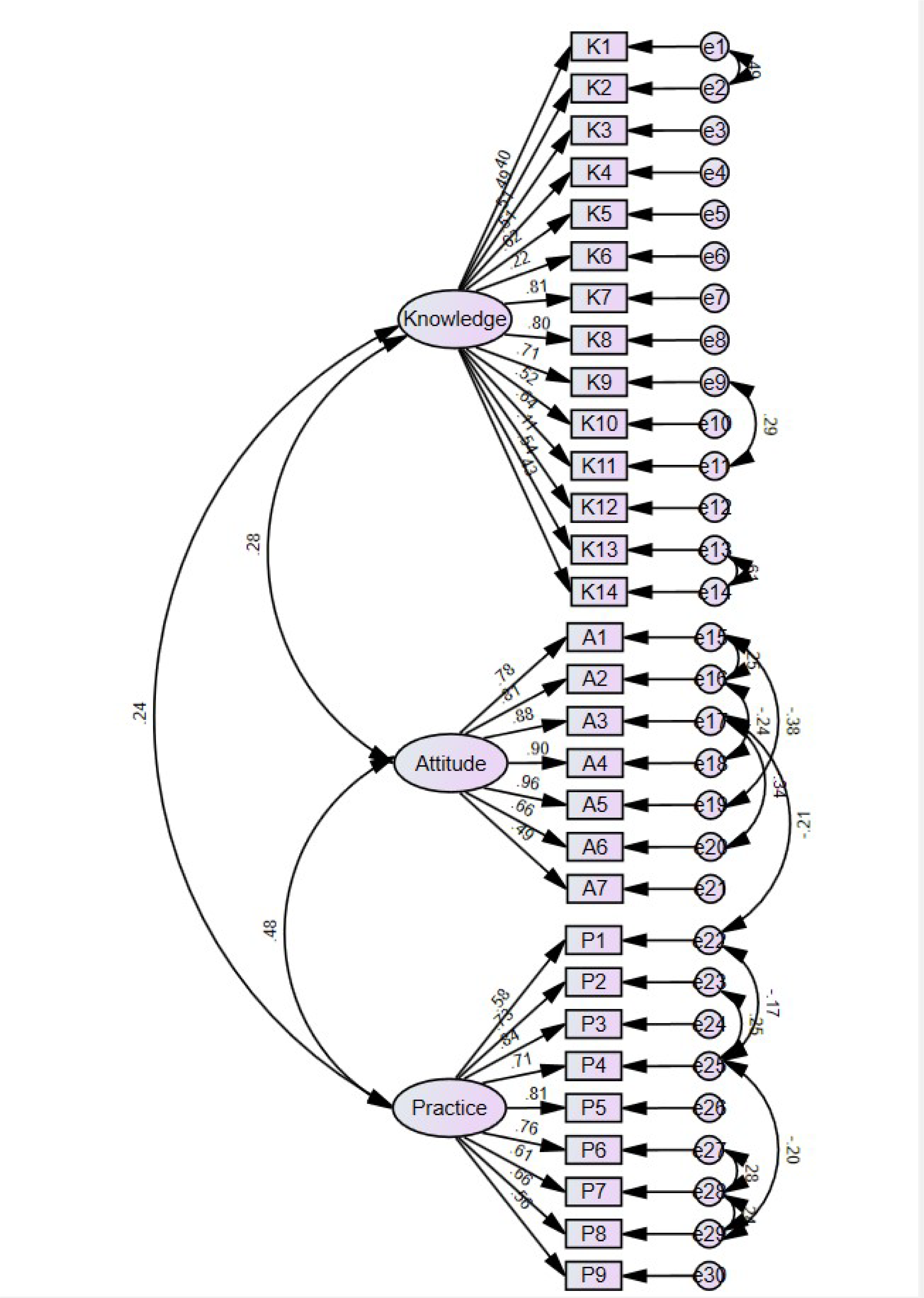

Supplement: SUPPLEMENTARY FIGURE S1 — Confirmatory factor analysis model. [file Image_1.tif]

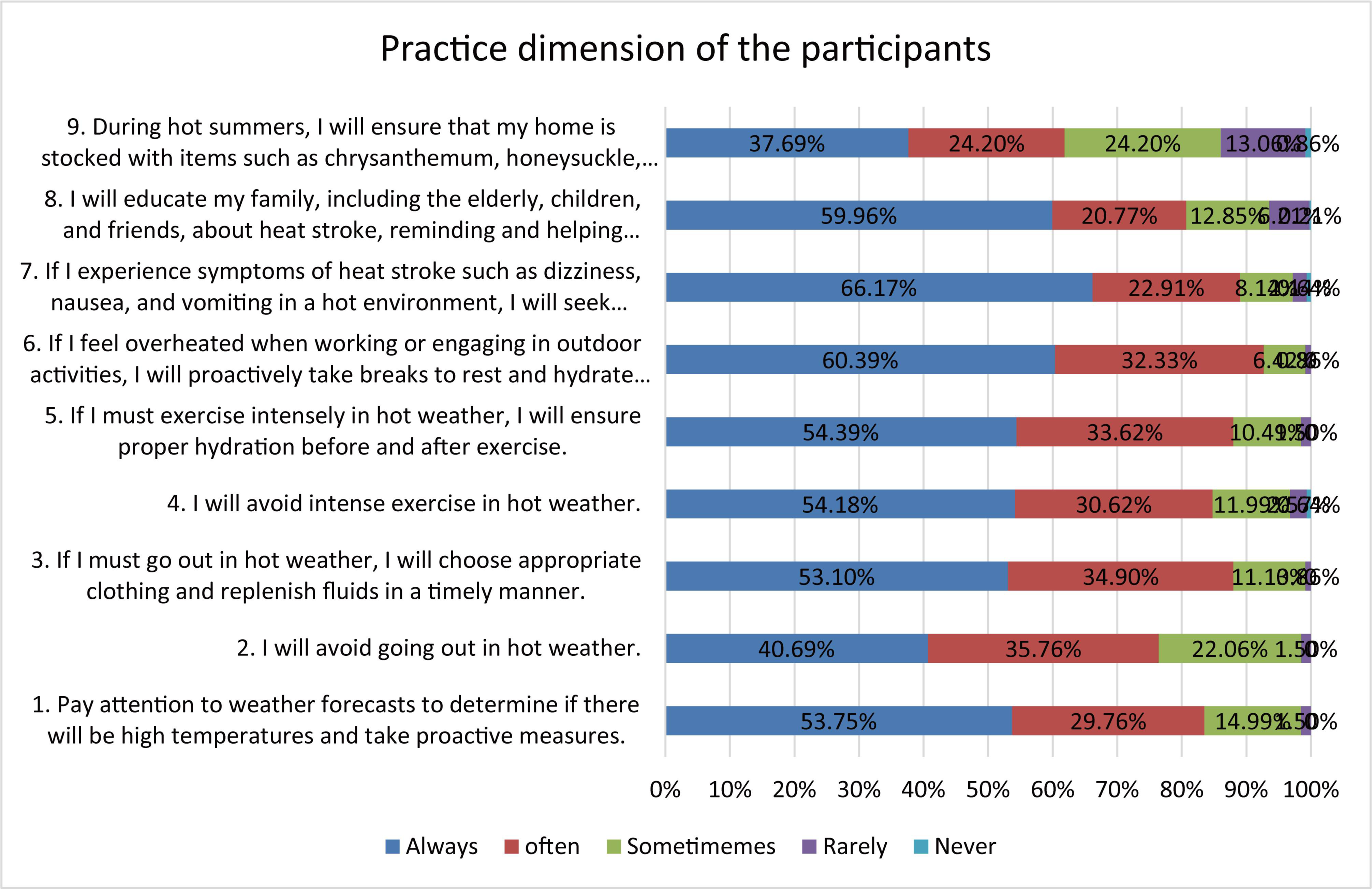

Supplement: SUPPLEMENTARY FIGURE S2 — Participants' attitude, practice dimension of each item option distribution. (A) attitude; (B) practice. [file Image_2.tif]
