# Supplementary material for: Public knowledge, attitudes, and practices toward heat stroke in Ningbo, China: a cross-sectional study
Source: Front Public Health. 2025 Sep 25;13:1659132. doi: 10.3389/fpubh.2025.1659132 (PMC12507894; doi:10.3389/fpubh.2025.1659132)
Supplement: Supplementary file 3 [file Table_1.docx]

Table S1: Factor Loadings from Confirmatory Factor Analysis

| Indicators Actual  CMIN/DF 4.394  RMSEA 0.085  IFI 0.838  TLI 0.818  CFI 0.837 | | | **Estimate** | **S.E.** | **C.R.** | **P** |
| --- | --- | --- | --- | --- | --- | --- |
| K1 | <--- | Knowledge | 1 |  |  |  |
| K2 | <--- | Knowledge | 1.111 | 0.115 | 9.663 | *** |
| K3 | <--- | Knowledge | 1.096 | 0.155 | 7.072 | *** |
| K4 | <--- | Knowledge | 1.103 | 0.155 | 7.117 | *** |
| K5 | <--- | Knowledge | 1.339 | 0.173 | 7.73 | *** |
| K6 | <--- | Knowledge | 0.578 | 0.141 | 4.107 | *** |
| K7 | <--- | Knowledge | 1.206 | 0.144 | 8.389 | *** |
| K8 | <--- | Knowledge | 1.297 | 0.155 | 8.357 | *** |
| K9 | <--- | Knowledge | 1.021 | 0.127 | 8.054 | *** |
| K10 | <--- | Knowledge | 0.833 | 0.116 | 7.196 | *** |
| K11 | <--- | Knowledge | 0.983 | 0.126 | 7.773 | *** |
| K12 | <--- | Knowledge | 0.181 | 0.085 | 2.126 | 0.034 |
| K13 | <--- | Knowledge | 1.156 | 0.159 | 7.27 | *** |
| K14 | <--- | Knowledge | 0.923 | 0.141 | 6.531 | *** |
| A1 | <--- | Attitude | 1 |  |  |  |
| A2 | <--- | Attitude | 1.124 | 0.051 | 22.24 | *** |
| A3 | <--- | Attitude | 1.11 | 0.052 | 21.156 | *** |
| A4 | <--- | Attitude | 1.128 | 0.052 | 21.784 | *** |
| A5 | <--- | Attitude | 1.196 | 0.055 | 21.632 | *** |
| A6 | <--- | Attitude | 0.932 | 0.062 | 15.04 | *** |
| A7 | <--- | Attitude | 0.818 | 0.075 | 10.881 | *** |
| P1 | <--- | Practice | 1 |  |  |  |
| P2 | <--- | Practice | 1.299 | 0.106 | 12.226 | *** |
| P3 | <--- | Practice | 1.325 | 0.099 | 13.416 | *** |
| P4 | <--- | Practice | 1.294 | 0.116 | 11.138 | *** |
| P5 | <--- | Practice | 1.297 | 0.099 | 13.054 | *** |
| P6 | <--- | Practice | 1.089 | 0.087 | 12.586 | *** |
| P7 | <--- | Practice | 1.04 | 0.097 | 10.721 | *** |
| P8 | <--- | Practice | 1.354 | 0.119 | 11.409 | *** |
| P9 | <--- | Practice | 1.33 | 0.132 | 10.085 | *** |

Table S2. Factors of practice based univariable and multivariable logistic regression.

| Practice (A cutoff of 80% of the total score was used, with scores above 38 considered as positive practice, totaling 289 individuals; scores below 38 were considered as negative practice, totaling 178 individuals.) | Univariate logistic regression | | Multivariate logistic regression | |
| --- | --- | --- | --- | --- |
|  | OR (95%CI) | P | OR (95%CI) | P |
| Knowledge Dimension | 1.104 (1.039-1.174) | 0.001 | 1.027 (0.949-1.112) | 0.511 |
| Attitude Dimension | 1.550 (1.404-1.710) | <0.001 | 1.555 (1.404-1.724) | <0.001 |
| Age |  |  |  |  |
| 18-45 years | 1.425 (0.842-2.409) | 0.187 |  |  |
| 46-69 years | ref |  |  |  |
| BMI |  |  |  |  |
| Less than 18 | 4.333 (1.437-13.068) | 0.009 | 4.135 (1.202-14.223) | 0.024 |
| 18-24 | 1.011 (0.674-1.517) | 0.956 | 0.827 (0.503-1.360) | 0.455 |
| Greater than or equal to 24 | ref |  | ref |  |
| Gender |  |  |  |  |
| Male | 0.732 (0.499-1.076) | 0.112 |  |  |
| Female | ref |  |  |  |
| Marital Status |  |  |  |  |
| Married | 0.522 (0.347-0.785) | 0.002 | 0.433 (0.258-0.726) | 0.001 |
| Other | ref |  | ref |  |
| Residence |  |  |  |  |
| Urban | 2.344 (1.314-4.184) | 0.004 | 1.759 (0.851-3.634) | 0.127 |
| Rural | ref |  | ref |  |
| Education Level |  |  |  |  |
| High school/technical school and below | 0.283 (0.128-0.626) | 0.002 | 0.294 (0.112-0.775) | 0.013 |
| Junior college | 0.370 (0.172-0.798) | 0.011 | 0.340 (0.139-0.832) | 0.018 |
| Bachelor's degree | 0.437 (0.220-0.867) | 0.018 | 0.410 (0.186-0.906) | 0.027 |
| Master's degree or above | ref |  | ref | ref |
| Occupation |  |  |  |  |
| Occupations involving working in high-temperature environments | 0.629 (0.315-1.255) | 0.189 |  |  |
| Other occupations not involving working in high-temperature environments | ref |  |  |  |
| Average disposable income per capita in the family per year | 0.729 (0.488-1.088) | 0.122 |  |  |
| Less than 50,000 | ref |  |  |  |
| 50,000 or more |  |  |  |  |
| History of heat stroke or severe heat stroke |  |  |  |  |
| Yes | 0.544 (0.226-1.309) | 0.174 |  |  |
| No | ref |  |  |  |
| Presence of diabetes, cardiovascular disease, or other underlying diseases |  |  |  |  |
| With underlying diseases |  |  |  |  |
| Without any diseases | 0.694 (0.385-1.254) | 0.226 |  |  |
| Knowledge Dimension | ref |  |  |  |

**Table S3 Specific Effects of Each Path in the Structural Equation Model**

| Path | | | Estimate | S.E. | C.R. | P |
| --- | --- | --- | --- | --- | --- | --- |
| Attitude | <--- | Knowledge | .444 | .101 | 4.397 | *** |
| Practice | <--- | Knowledge | .323 | .125 | 2.588 | .010 |
| Practice | <--- | Attitude | .652 | .100 | 6.518 | *** |
| K1 | <--- | Knowledge | 1.000 |  |  |  |
| K2 | <--- | Knowledge | 1.134 | .121 | 9.382 | *** |
| K3 | <--- | Knowledge | 1.121 | .164 | 6.844 | *** |
| K4 | <--- | Knowledge | 1.126 | .164 | 6.879 | *** |
| K5 | <--- | Knowledge | 1.371 | .184 | 7.454 | *** |
| K6 | <--- | Knowledge | .569 | .146 | 3.907 | *** |
| K7 | <--- | Knowledge | 1.262 | .156 | 8.099 | *** |
| K8 | <--- | Knowledge | 1.330 | .166 | 8.032 | *** |
| K9 | <--- | Knowledge | 1.106 | .140 | 7.884 | *** |
| K10 | <--- | Knowledge | .867 | .124 | 7.010 | *** |
| K11 | <--- | Knowledge | 1.080 | .141 | 7.682 | *** |
| K12 | <--- | Knowledge | .170 | .088 | 1.931 | .053 |
| K13 | <--- | Knowledge | 1.190 | .169 | 7.046 | *** |
| K14 | <--- | Knowledge | .943 | .149 | 6.334 | *** |
| A7 | <--- | Attitude | 1.000 |  |  |  |
| A6 | <--- | Attitude | 1.135 | .104 | 10.965 | *** |
| A5 | <--- | Attitude | 1.519 | .133 | 11.454 | *** |
| A4 | <--- | Attitude | 1.416 | .127 | 11.189 | *** |
| A3 | <--- | Attitude | 1.360 | .123 | 11.035 | *** |
| A2 | <--- | Attitude | 1.372 | .129 | 10.673 | *** |
| A1 | <--- | Attitude | 1.171 | .114 | 10.253 | *** |
| P1 | <--- | Practice | 1.000 |  |  |  |
| P2 | <--- | Practice | 1.347 | .112 | 12.022 | *** |
| P3 | <--- | Practice | 1.337 | .104 | 12.802 | *** |
| P4 | <--- | Practice | 1.309 | .113 | 11.613 | *** |
| P5 | <--- | Practice | 1.329 | .105 | 12.657 | *** |
| P6 | <--- | Practice | 1.135 | .092 | 12.360 | *** |
| P7 | <--- | Practice | 1.138 | .103 | 11.025 | *** |
| P8 | <--- | Practice | 1.376 | .124 | 11.126 | *** |
| P9 | <--- | Practice | 1.333 | .137 | 9.752 | *** |
