# Supplementary material for: Public knowledge, attitudes, and practices toward heat stroke in Ningbo, China: a cross-sectional study
Source: Front Public Health. 2025 Sep 25;13:1659132. doi: 10.3389/fpubh.2025.1659132 (PMC12507894; doi:10.3389/fpubh.2025.1659132)
Supplement: Supplementary file 4 [file Table_2.docx]

Questionnaire Number:

| Dear friend,  We are researchers from ** Hospital, and we sincerely invite you to participate in our research project. This study aims to understand the public's knowledge, attitudes, and practices towards heatstroke, in order to provide a basis for developing scientific early intervention strategies. This may help more people in the future improve their health. Your participation in this study is voluntary. If you agree to participate in this study, please refer to the following instructions.  1. Please complete the questionnaire. There are no right or wrong answers. You only need to fill in according to the actual situation. You can ask us any questions during the answering process. Please submit it promptly after completion.  2. This study is a simple questionnaire survey, which will not cause harm to your physical and mental health. However, it will involve some privacy issues, such as your gender, age, etc. We will strictly protect your confidentiality and will not disclose your information. Please feel free to fill it out.  3. As a participant, you can always understand the information and research progress related to this study. If you decide to withdraw from the study, please inform us. Your data will not be included in the research results.  Finally, we sincerely thank you for taking the time to support our scientific research amidst your busy schedule!  □ I am aware of and agree to the use of the collected data for scientific research.  Informed Consent Signature:  Date of Participation: Year Month |
| --- |

| **Part 1 Basic Information** | |
| --- | --- |
| 1. **Your age: years** | |
| 1. **Your height: cm** | |
| 1. **Your weight: kg (1 kg = 2 catties)** | |
| 1. **Your gender: a. Male b. Female** |  |
| 1. **Your marital status:** | A. Unmarried  B. Married  C. Divorced  D. Widowed |
| 1. **Your place of residence:** | a. Urban  b. Rural |
| 1. **Your city/region of residence (can be set via Questionnaire Star country/region settings):** | Province |
| 1. **Your educational background:** | a. Primary school and below  b. Junior high school  c. High school/Technical secondary school  d. Junior college  e. Bachelor's degree  f. Master's degree and above |
| 1. **Your occupation:** | a. Sanitation worker  b. Traffic police  c. Firefighter  d. Athlete/Sports coach  e. Construction worker  f. Other jobs involving high-temperature environments  g. Other jobs not involving high-temperature environments |
| 1. **Average disposable income per person in your household per year: (RMB)** | a. Less than 20,000 RMB  b. 20,000-30,000 RMB  c. 30,000-40,000 RMB  d. 40,000-50,000 RMB  e. Greater than or equal to 50,000 RMB |
| 1. **Have you ever experienced heatstroke/ heat illness?** | a. Yes  b. No |
| 1. **Do you have the following diseases?** |  |
| **13.1 Diabetes** | a. Yes  b. No |
| **13.2 Cardiovascular disease** | a. Yes  b. No |
| **13.3 Other underlying diseases: hyperthyroidism, schizophrenia, Parkinson's disease, hypohidrosis, severe skin diseases.** | a. Yes  b. No |

| **Part 2: Understanding of Heatstroke Knowledge** | | | |
| --- | --- | --- | --- |
| **Please judge the following statements based on your existing knowledge. If you are unsure, you can select the "Not sure" option.** | | | |
| **1. Heatstroke, also known as severe heat illness, is divided into classic heatstroke and exertional heatstroke, often occurring in humid and hot conditions.** | a. Yes | b. No | c. Not sure |
| **2. Classic heatstroke is due to impaired heat dissipation caused by dysfunction in temperature regulation in high-temperature environments.** | a. Yes | b. No | c. Not sure |
| **3. Classic heatstroke mainly occurs in the elderly, children, and individuals with underlying diseases.** | a. Yes | b. No | c. Not sure |
| **4. Inadequate intake of water to replenish fluid loss caused by sweating can lead to dehydration, which is an important factor in the occurrence of non-exertional heatstroke.** | a. Yes | b. No | c. Not sure |
| **5. Exertional heatstroke typically occurs in previously healthy young individuals who engage in vigorous exercise in high ambient temperatures and humidity.** | a. Yes | b. No | c. Not sure |
| **6. Heatstroke cannot occur during exercise in cool environments.** | a. Yes | b. No | c. Not sure |
| **7. Symptoms of heatstroke can include dizziness, nausea, muscle cramps, and confusion.** | a. Yes | b. No | c. Not sure |
| **8. In clinical settings, heatstroke can manifest as central nervous system dysfunction and multi-organ system failure.** | a. Yes | b. No | c. Not sure |
| **9. If not promptly treated, heatstroke can result in death.** | a. Yes | b. No | c. Not sure |
| **10. The core body temperature of heatstroke patients can rise to over 40°C.** | a. Yes | b. No | c. Not sure |
| **11. Rapid, effective, and sustained cooling is the primary treatment for heatstroke.** | a. Yes | b. No | c. Not sure |
| **12. The use of medications such as antipyretics to reduce fever may be necessary in the treatment of heatstroke.** | a. Yes | b. No | c. Not sure |
| **13. Heatstroke patients may experience involuntary muscle spasms.** | a. Yes | b. No | c. Not sure |
| **14. Common herbs such as chrysanthemum, honeysuckle, lotus leaf, peppermint, patchouli, and pogostemon cablin have heat-clearing and heatstroke-preventing effects to some extent.** | a. Yes | b. No | c. Not sure |

| **Part 3: Attitudes Towards Heatstroke** | |
| --- | --- |
| **1. I believe heatstroke is a serious health issue that should be given sufficient attention by the public.** | A. Strongly Agree  B. Agree  C. Neutral  D. Disagree  E. Strongly Disagree |
| **2. I believe public places should provide adequate heatstroke prevention facilities to reduce the risk of heatstroke.** | A. Strongly Agree  B. Agree  C. Neutral  D. Disagree  E. Strongly Disagree |
| **3. I believe the government should take measures to educate the public on how to prevent heatstroke.** | A. Strongly Agree  B. Agree  C. Neutral  D. Disagree  E. Strongly Disagree |
| **4. I believe households should be equipped with necessary heatstroke prevention measures to protect family members from heatstroke.** | A. Strongly Agree  B. Agree  C. Neutral  D. Disagree  E. Strongly Disagree |
| **5. I believe it is necessary to focus on educating high-risk groups for heatstroke such as the elderly, children, and workers about heatstroke.** | A. Strongly Agree  B. Agree  C. Neutral  D. Disagree  E. Strongly Disagree |
| **6. I believe it is important to prevent heatstroke in advance to reduce the mortality rate of heatstroke.** | A. Strongly Agree  B. Agree  C. Neutral  D. Disagree  E. Strongly Disagree |
| **7.I am confident in preventing heatstroke.** | A. Strongly Agree  B. Agree  C. Neutral  D. Disagree  E. Strongly Disagree |

| **Part 4: Practices in Preventing Heatstroke** | |
| --- | --- |
| **1. Pay attention to weather forecasts to determine if there will be high temperatures and take proactive measures.** | A. Always  B. Often  C. Sometimes  D. Rarely  E. Never |
| **2. I will avoid going out in hot weather.** | A. Always  B. Often  C. Sometimes  D. Rarely  E. Never |
| **3. If I must go out in hot weather, I will choose appropriate clothing and replenish fluids in a timely manner.** | A. Always  B. Often  C. Sometimes  D. Rarely  E. Never |
| **4. I will avoid intense exercise in hot weather.** | A. Always  B. Often  C. Sometimes  D. Rarely  E. Never |
| **5. If I must exercise intensely in hot weather, I will ensure proper hydration before and after exercise.** | A. Always  B. Often  C. Sometimes  D. Rarely  E. Never |
| **6. If I feel overheated when working or engaging in outdoor activities, I will proactively take breaks to rest and hydrate to prevent heatstroke.** | A. Always  B. Often  C. Sometimes  D. Rarely  E. Never |
| **7. (This item appears to be missing.)** |  |
| **8. If I experience symptoms of heatstroke such as dizziness, nausea, and vomiting in a hot environment, I will seek medical help immediately.** | A. Always  B. Often  C. Sometimes  D. Rarely  E. Never |
| **9. I will educate my family, including the elderly, children, and friends, about heatstroke, reminding and helping them to prevent it.** | A. Always  B. Often  C. Sometimes  D. Rarely  E. Never |
| **10. During hot summers, I will ensure that my home is stocked with items such as chrysanthemum, honeysuckle, lotus leaf, mint, Agastache rugosa, and Pogostemon cablin, which are effective in cooling and relieving heat, and carry them when necessary.** | A. Always  B. Often  C. Sometimes  D. Rarely  E. Never |
